# Cellular Mechanism Underlying rTMS Treatment for the Neural Plasticity of Nervous System in *Drosophila* Brain

**DOI:** 10.3390/ijms20184625

**Published:** 2019-09-18

**Authors:** Ying Luo, Junqing Yang, Hong Wang, Zongjie Gan, Donzhi Ran

**Affiliations:** 1 Department of Pharmacology, Chongqing Medical University, Chongqing 400016, China; wolying@aliyun.com (Y.L.); cqyangjq@cqmu.edu.cn (J.Y.); 101832@cqmu.edu.cn (H.W.); gzj@cqmu.edu.cn (Z.G.); 2 The Key Laboratory of Biochemistry and Molecular Pharmacology, Chongqing Medical University, Chongqing 400016, China; 3 Chongqing Research Center for Pharmaceutical Engineering, Chongqing Medical University, Chongqing 400016, China

**Keywords:** repetitive transcranial magnetic stimulation, projection neurons, excitatory neuronal transmission, synaptic plasticity, calcium channel

## Abstract

Repetitive transcranial magnetic stimulation (rTMS) is used as a research tool and clinical treatment for the non-clinical and clinical populations, to modulate brain plasticity. In the case of neurologic and psychiatric disease, there is significant evidence to suggest that rTMS plays an important role in the functional recovery after neurological dysfunction. However, the causal role for rTMS in the recovery of nervous dysfunction remains unclear. The purpose of the present study is to detect the regulation of rTMS on the excitatory neuronal transmission and specify the mode of action of rTMS on the neural plasticity using *Drosophila* whole brain. Therefore, we identified the effects of rTMS on the neural plasticity of central neural system (CNS) by detecting the electrophysiology properties of projection neurons (PNs) from adult *Drosophila* brain after rTMS. Using patch clamp recordings, we recorded the mini excitatory postsynaptic current (mEPSC) of PNs after rTMS at varying frequencies (1 Hz and 100 Hz) and intensities (1%, 10%, 50%, and 100%). Then, the chronic electrophysiology recordings, including mEPSC, spontaneous action potential (sAP), and calcium channel currents from PNs after rTMS at low frequency (1 Hz), with low intensity (1%) were detected and the properties of the recordings were analyzed. Finally, the frequency and decay time of mEPSC, the resting potential and frequency of sAP, and the current density and rise time of calcium channel currents were significantly changed by rTMS. Our work reveals that rTMS can be used as a tool to regulate the presynaptic function of neural circuit, by modulating the calcium channel in a frequency-, intensity- and time-dependent manner.

## 1. Introduction 

Transcranial magnetic stimulation (TMS), known as repetitive transcranial magnetic stimulation (rTMS), is a non-invasive form of brain stimulation, which has been commonly used as a neurologic and psychiatric research tool and clinical treatment for diagnostic and therapeutic potential in the central nervous system (CNS). rTMS has been widely used in clinical treatment, such as schizophrenia [1], and chronic stroke [2], and has been regarded to be safe. However, adverse effects of rTMS have also been detected. It is reported that rTMS could lead to fainting and seizures [3]. Patients have the risks for cognitive dysfunction, pain, and memory loss after Rtms [4]. These works call our attention to the mechanisms of rTMS and its contributions to diverse clinical outcomes. 

rTMS results in varieties of effects depending on the stimulation parameters, including intensity and frequency. Either low-frequency [5] or high-frequency [6] is reported to improve the neural activities; however, opposite effects of low-frequency and high-frequency have also been detected [5,7]. Additionally, numerous researchers tried to uncover the relation between varying rTMS intensities and neural circuit activities [8,9]. Although many studies have been reported, there are still some doubts about the most effective parameters of stimulation. Particularly, it is ambiguous that how rTMS with different intensities, at different frequencies, can modulate the neural circuit activities, such as the neural plasticity which is one of the most important characters of the neural function, because of the limitation of animal models. Cultured neurons [10,11] and hippocampal slices [12] from rats and even humans [13,14] have been utilized to study the role of rTMS. However, these models are difficult to present a complete loop neural circuit. The fruit fly, *Drosophila* melanogaster, has been used as an ideal model to study the characteristics of neural function because of the similarity of nervous systems between fruit flies and human beings [15,16]. In addition, the simple neural network and well-characterized neurons provide a useful mean for presenting the changes of neural function. Projection neurons (PNs), as a second olfactory neuron of *Drosophila* CNS, play a key role on the formation of neural networks. PNs are divided into multiple classes by glomerulus and receive inputs from the local neurons (LNs), with PNs dendrite targeting the higher olfactory region, consisting of Kenyon cells (KCs), forming a LNs-PNs-KCs loop [17,18,19]. Thus, the changes of PNs can show the PN’s real reaction to stimulation and present the changes of LNs-PNs-KCs loop. Therefore, unlike other animal model, including neuron cultures which always shows the changes of a single neuron, and rat brain slices which always show the changes of part of the neural circuit, the whole brain of *Drosophila* can offer us an ideal animal model with LNs-PNs-KCs loop, to learn the true response of intact neural loop, to detect directly the neural plasticity of PNs, and to get the acute and chronic effect of rTMS. 

In our study, we detected the effects of rTMS at varying frequencies and varying intensities on PNs utilizing the patch clamp recordings from isolated whole brain of *Drosophila* model, which provided an intact neural circuit, showing the genuine response to rTMS. As a marker of the neural plasticity of excitatory synapse, mini excitatory postsynaptic current (mEPSC) of PNs was detected after the rTMS at different frequencies (1 Hz and 100 Hz) and intensities (1%, 10%, 50%, and 100%), indicating that rTMS regulated the neural plasticity in a frequency- and intensity-dependent manner. Then, the acute (immediately after rTMS) and chronic (12 h and 24 h after rTMS, shown as 12-h and 24-h) effects of rTMS at low-frequency (1-Hz) and low-intensity (1%) on mEPSC and spontaneous action potential (sAP) are detected to show that the properties of neural circuit changed by the rTMS in a time-dependent manner. Finally, the calcium channel activities from PNs are shown, releasing the potential cellular mechanisms of rTMS -induced neural plasticity activities.

## 2. Results

### 2.1. The Effects of rTMS (at 1 Hz, with 1%, 10%, 50%, and 100% Intensities, and at 1 Hz, 2 Hz, 5 Hz, and 10 Hz, with 1% Intensity) on the mEPSC of PNs

To assess the neural circuit responses to rTMS, the acute and chronic effects of rTMS on the electrophysiology properties of PNs from the whole brains of *Drosophila* were detected. The cell bodies of the same class of PNs, which aggregate in the glomerulus of the antennal lobe (AL), respond to the specific patterns of the stimulation [20]. In the AL, PNs from the same glomeruli accept the information from LNs and transfer it to the higher neurons of KCs, which are located in the mushroom body (MB). These neurons, including LNs, PNs, and KCs, form a neuronal circuit, meanwhile the PNs responses are modulated by the properties of LN-to-PN synapses [21]. As a key and second-order olfactory neuron of *Drosophila* neural circuit, it is of importance to estimate the synaptic transmission of PNs. 

**Figure 1:**
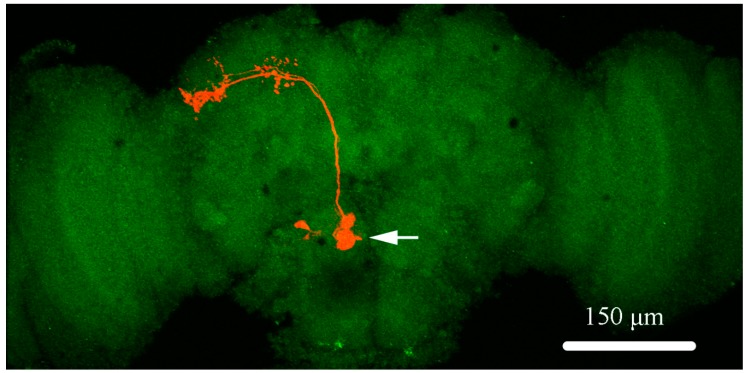
The schematic presentation of projection neurons (PNs) in *Drosophila* brain. The morphology properties of a single PN (in orange) is labeled with biocytin. The soma (marked by a white arrow) of the PN, located in the antennal lobe region in discrete glomeruli, and the major branch of PN, giving off several small collaterals, projected dorso-medially in isolated brain.

**Figure 2:**
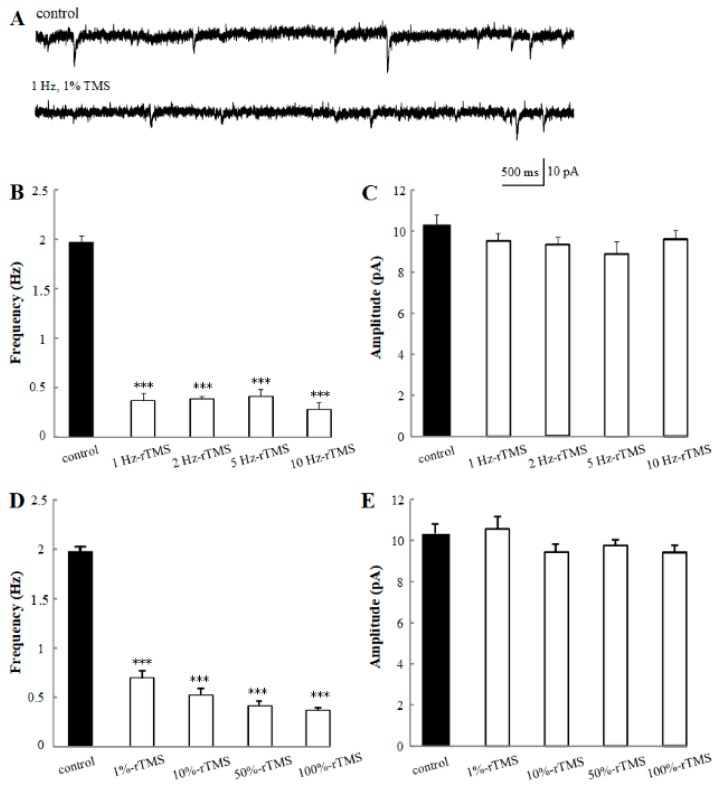
Effects of repetitive transcranial magnetic stimulation (rTMS) at varying frequencies and intensities on the mini excitatory postsynaptic current (mEPSC) recorded from the PNs of isolated *Drosophila* brain. (**A**) Representative traces of the mEPSC from control and 1 Hz, 1% rTMS groups. (**B**,**C**) show the mean frequencies and amplitudes of the mEPSC recorded from PNs with 100% rTMS at different frequencies (1 Hz, 2 Hz, 5 Hz, and 10 Hz). (**D**,**E**) show the mean frequencies and amplitudes of the mEPSC recorded from PNs after rTMS at 1 Hz with different intensities (1%, 10%, 50%, and 100%) TMS and control group, respectively. Data are expressed as mean ± SEM (Standard Error of Mean). (*** *p* means < 0.001 compared with the control group; *n* = 6 in each group).

Thus, above all, the morphological properties of PNs in an isolated brain are shown in Figure 1. The soma of PNs, which is the exact spatial sites of pipettes, is always easily visible under the microscope, since it is located on the top of AL and stays suspended in the external solution. Then, the electrophysiological properties of PNs have been recorded and analyzed. First, we detected the potential regulation of rTMS at low and high frequency ranging from 1 Hz to 10 Hz, with 100% intensity on mEPSC. The amplitude and frequency of mEPSC were analyzed (Figure 2). The average amplitude and frequency of control group are 10.30 ± 0.43 pA and 1.91 ± 0.11 Hz. With the application of 100% rTMS at 1 Hz, 2 Hz, 5 Hz, and 10 Hz, the frequencies were significantly changed to 0.36 ± 0.07 Hz (1 Hz-rTMS, *p* < 0.001), 0.39 ± 0.03 Hz (2 Hz-rTMS, *p* < 0.001), 0.43 ± 0.07 Hz (5 Hz-rTMS, *p* < 0.001), and 0.27 ± 0.06 Hz (10 Hz-rTMS, *p* < 0.001), respectively; however, the amplitude did not change. Our data showed that either low or high frequency rTMS could decrease the frequency, but not the amplitude of mEPSC of PNs, suggesting a regulation of rTMS on the mEPSC of PNs in a frequency-dependent manner. Second, the effects of rTMS with varying intensities (1%, 10%, 50%, and 100%) were detected. The average mEPSC frequencies significantly decreased to 0.70 ± 0.07 Hz (1%-rTMS, *p* < 0.001), 0.53 ± 0.04 Hz (10%-rTMS, *p* < 0.001), 0.42 ± 0.05 Hz (50%-rTMS, *p* < 0.001), and 0.36 ± 0.07 Hz (100%-rTMS, *p* < 0.001) after TMS, compared with the control group (1.91 ± 0.11 Hz); however, the mEPSC amplitude did not change, suggesting a regulation of rTMS on the mEPSC of PNs in an intensity-dependent manner. Our data indicated that rTMS affects the mEPSC properties of PNs in a frequency- and intensity-dependent manner.

### 2.2. The Acute and Chronic Effects of 100% rTMS at 1 Hz on the mEPSC from PNs

**Figure 3:**
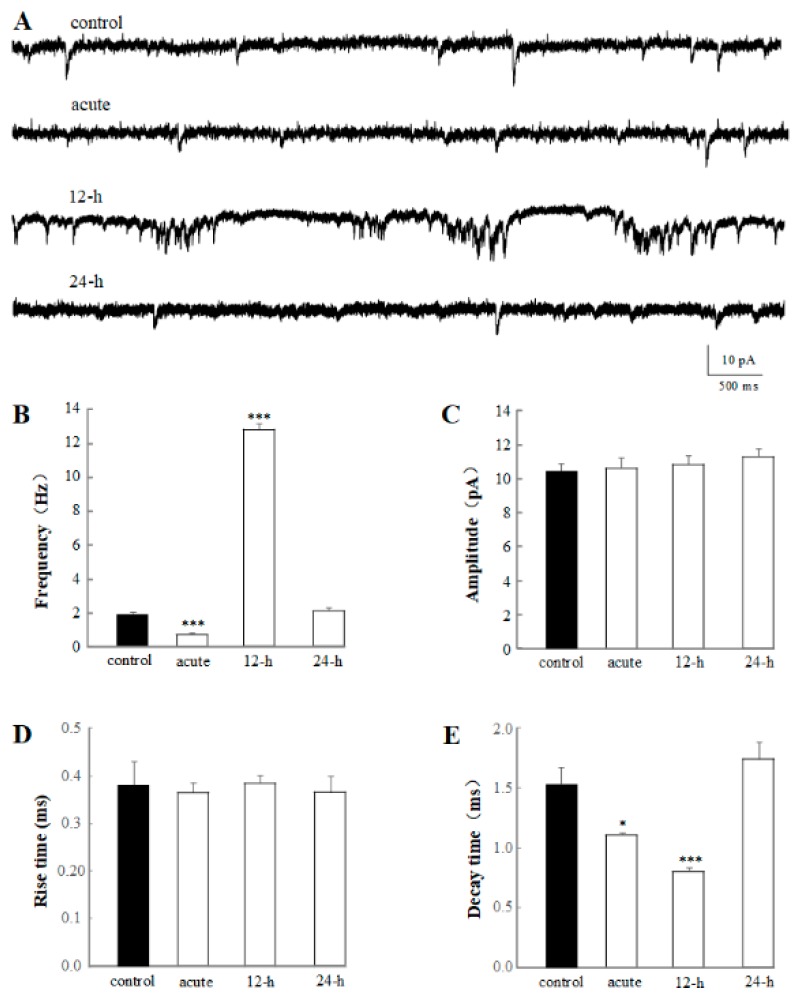
Acute and chronic effects of 1% rTMS at 1 Hz, on the mEPSC recorded from PNs of isolated *Drosophila* brain. (**A**) shows the representative traces of mEPSC from control, acute, 12-h, and 24-h groups. (**B**–**E**) show the average frequencies, amplitude, rise time, and decay time of mEPSC recorded from PNs with 1% rTMS and control group, respectively. Data are expressed as mean ± SEM. (*p means < 0.05, *** *p* < 0.001 compared with the control group; *n* = 6 in each group).

In the present experiment, significant decrease in mEPSC frequency have been detected in the application of varying test rTMS frequencies and intensities, showing the acute effect of rTMS in a frequency- and intensity-dependent manner (Figure 2). To further determine the alteration of rTMS in mEPSC, the chronic effects of 1% rTMS were recorded, and the average frequency, amplitude, rise time, and decay time of mEPSC were analyzed (Figure 3). The representative traces of mEPSC of control, acute, 12-h group, and 24-h group are shown in Figure 3A. The frequencies but not the amplitudes were altered. Compared to the control group (1.91 ± 0.11 Hz), the mEPSC frequency of acute group (0.70 ± 0.073 Hz, *p* < 0.001) decreased; however, the 12-h group (12.67 ± 0.33 Hz, *p* < 0.001) increased significantly, showing a burst of mEPSC (Figure 3B). The decay times of mEPSC in acute group (1.12 ± 0.017 ms, *p* < 0.05) and 24-h group (0.80 ± 0.030 ms, *p* < 0.001) reduced significantly, compared with the control group (1.52 ± 0.13 ms, Figure 3E). However, no difference was detected in mEPSC amplitude and rise time (Figure 3C,D). According to our data, the average frequency, amplitude, rise time, and decay time of the 24-h group seemed to have no difference with the control group (Figure 3), indicating a regulation of rTMS on the mEPSC of PNs in a time-dependent manner.

### 2.3. The sAP Is Significantly Affected by 1% rTMS at 1 Hz

**Figure 4:**
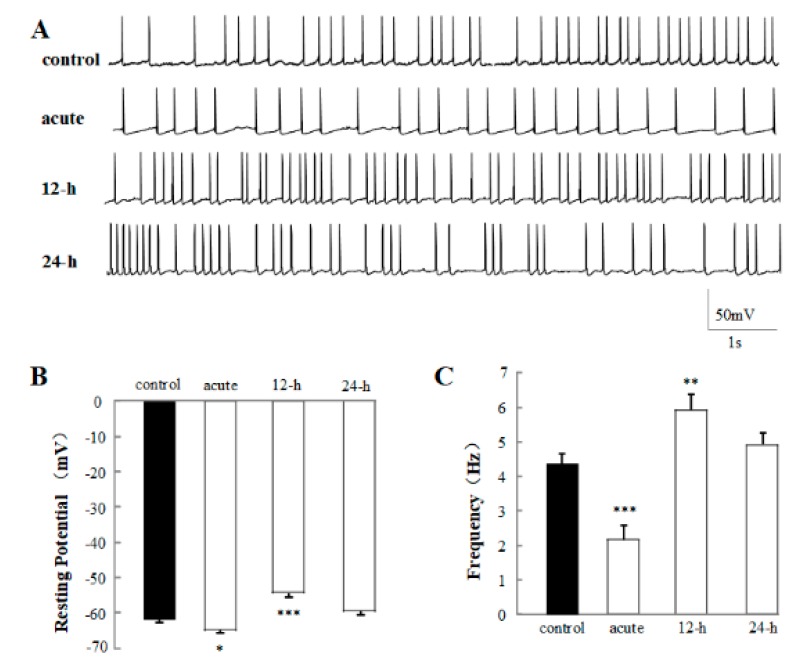
Effects of 1% rTMS at 1 Hz, on the spontaneous action potential (sAP) recorded from PNs of isolated *Drosophila* brain. (**A**) shows the representative traces of sAP of control, acute, 12-h, and 24-h groups. (**B**,**C**) show the average resting potential and the frequency of sAP, recorded from PNs with 1% rTMS group and control group, respectively. Data are expressed as mean ± SEM. (*p means < 0.05, **p means < 0.01, ***p < 0.001, compared with the control group; *n* = 6 in each group).

In order to uncover the effects of 1% rTMS at 1 Hz on the excitability of neurons in the neural circuit, the sAPs of the PNs were recorded at the current-clamp voltage. The representative traces of sAP of control, acute, 12-h group, and 24-h group are shown in Figure 4A. The average resting potential (RP) (Figure 4B), frequency (Figure 4C) were analyzed. The average RP and frequency of control group were −62.40 ± 0.81 ms and 4.33 ± 0.30 Hz, respectively. After the application of 1% rTMS at 1 Hz, the average RP of acute group increased to −65.20 ± 1.11 mV (*p* < 0.05), and the frequency decreased to 2.22 ± 0.41 Hz (*p* < 0.001). The average RP of 12-h group decreased to −54.40 ± 0.93 mV (*p* < 0.001), and the frequency increased to 5.94 ± 0.43 Hz (*p* < 0.01). No significant RP and frequency changes were detected in the 24-h group. This data showed the effects of rTMS on the excitability of the PNs.

### 2.4. The Calcium Channel Current Is Significantly Affected by 1% rTMS at 1 Hz

**Figure 5:**
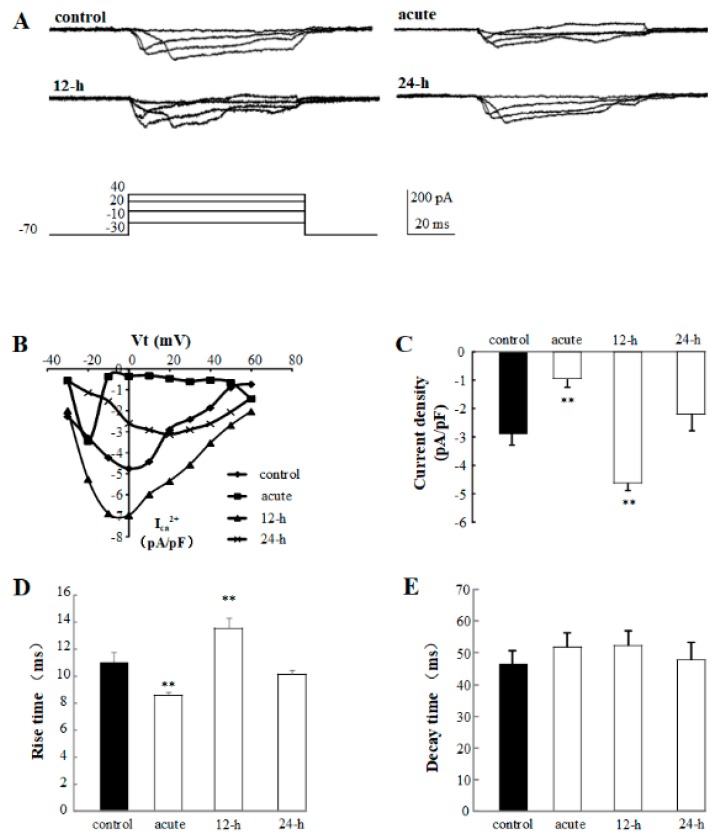
Effects of 1% rTMS at 1 Hz on the calcium channel activities recorded from PNs of isolated *Drosophila* brain. (**A**) shows the representative traces of the calcium channel activities of control, acute, 12-h, and 24-h groups. (**B**) The I-V curves show the calcium current densities evoked by current injections. (**C**) shows the average current densities of PNs from control group, acute, 12-h, and 24-h groups. (**D**,**E**) show the average rise times and decay times of calcium channel activities recorded from PNs of control group, acute, 12-h, and 24-h groups, respectively. (**p means < 0.01, compared with the control group; *n* = 6 in each group).

To identify the cellular mechanism of rTMS-induced neural plasticity changes, the calcium channel current was detected. The representative traces of calcium channel of control, acute, 12-h, and 24-h groups are shown in Figure 5A. From the I-V curve in Figure 5B, we can notice that the calcium channel current density can be modulated by rTMS. Our data show that the calcium current density of acute group is inhibited to −0.94 ± 0.31 pA/pF (*p* < 0.01), and the 12-h group is activated to −4.63 ± 0.60 pA/pF (*p* < 0.01) compared with the control group (−2.86 ± 0.45 pA/pF), but no significant change has been observed in the 24-h group (Figure 5C). The rise time and decay time were analyzed. Compared to the control group (11.01 ± 0.69 ms), the rise time of acute and 12-h groups changed to 8.62 ± 0.18 ms (*p* < 0.01) and 13.58 ± 0.72 ms (*p* < 0.01), respectively, but the 24-h group has not changed (Figure 5D). There is no difference in the decay time among these four groups (Figure 5E). In conclusion, the regulation of rTMS on the electrophysiological properties of PNs, including the neural plasticity and excitability of PNs, have shown to be associated with calcium channel activities in a time-dependent manner.

## 3. Discussion

rTMS is a potential method for the treatment of neurological and psychiatric disorders, by altering and modulating the activities of neural circuit. Meanwhile, the beneficial effects of rTMS in improving brain function have been reported [1,2,22] using animal models. However, the physiological bases of rTMS after-effects have not yet been clearly identified, and the long-term and short-term effects of rTMS at different frequencies and intensities on the intact neural circuit have not been clearly shown simultaneously. Thus, the present study meant to test the effects of rTMS with varying intensities and frequencies on the electrophysiology properties of neurons in a comprehensive inspection; additionally, acute and chronic effects have also been examined and analyzed. 

As an ideal model in neuroscience research, the *Drosophila* model has been used in the present study. Unlike culture cells and rat brain slices, our whole *Drosophila* brain provided an excellent system to show the activity of the intact neural circuit loop. The electrophysiology characteristics of the PNs from the *Drosophila* olfactory circuit, which investigate the relationship between stimulations and the response of neural circuit, have been detected [23]. In this neural circuit, PNs occupies an important position. PNs synapse with the primary neuron in the antennal lobe send their axons to KCs [18]. Thus, PNs are mainly involved in the input and output activities of LNs and KCs, and exhibit distinguishable electrophysiological properties and responses, contributing to the information process, cognitive, learning and memory, accompanied by the changes of ion channels. Synaptic plasticity has the ability to improve the function of the brain network, enable short- and long-term remodeling of neural communication [24]. In this study, we focused on the synaptic plasticity to uncover the effects of rTMS on the brain function. We mainly detected the mEPSC of PNs, which is recorded to measure the synaptic plasticity. 

There are many rTMS protocols, which can lead to different lasting effects. Stimulus frequency, stimulus intensity, duration of the application, and the number of stimuli are the variables influencing factors [25]. However, the effects of each factor are controversial. It has reported that the low-frequency stimulation (≤ 1Hz) has inhibitory effects on neurons; however, the high-frequency stimulation (> 5Hz) has excitatory effects [26]. To solve this question, the regulation of rTMS at varying frequencies, with varying intensities have been estimated, using our ideal intact loop circuit model. In the first part, the mEPSC of PNs from isolated *Drosophila* brain after 100% rTMS at 1 Hz, 2 Hz, 5 Hz, and 10 Hz was detected (Figure 2B,C). Second, the mEPSC was recorded after rTMS at 1 Hz with 1%, 10%, 50%, and 100% (Figure 2D,E). In these two recordings, the frequencies but not the amplitudes of mEPSC decreased significantly by rTMS no matter with low- or high- intensities, at low- or high- frequencies. Thus, we concluded that rTMS (with low- or high- intensities, at low- or high- frequencies) can inhibit the mEPSC, and the synaptic plasticity is regulated by rTMS in a frequency- and intensity-dependent manner. mEPSC, which is one of the most important features of neurons, has been used to show the synaptic plasticity. It has been widely known that the frequency of mEPSC usually presents the properties of pre-synaptic synapses, including the number of neurotransmitter release regulated by calcium channel, while the amplitude of mEPSC reflects the interactions between neurotransmitter and receptors [27]. Therefore, according to our data, we can say that the properties of pre-synaptic synapses, for example, the activities of calcium channel which associate with the release of vesicles, have been influenced by rTMS in a frequency- and intensity-dependent manner.

We should note that one main purpose of this research is to study the acute and chronic effects of rTMS, as few long-lasting effects of rTMS on neural circuit has been reported, because of the limitation of animal models or some other reasons. Here, we focused on the properties of mEPSC (Figure 3), sAP (Figure 4), and calcium channel (Figure 5), 12 h and 24 h after 1% rTMS at 1 Hz. First, the mEPSC was recorded and estimated (Figure 3). The amplitudes and rise times of mEPSC from the four groups did not change. The decay times of mEPSC in acute and 12-h groups decreased compared to the control group. Interestingly, the frequency of the acute group decreased significantly compared to the control group, however, the 12-h group increased, and the 24-h group returned to average level. These results indicated that rTMS would affect the pre-synaptic in a time-dependent manner. 

Consequently, in acute and chronic experiments, the authors observed that the frequency, but not amplitude of mEPSC was changed significantly by rTMS in a frequency-, intensity- and time-dependent manner, indicating the potential role of rTMS in the synaptic plasticity and neural circuit. 

In the next part, sAP and calcium channel have been recorded to detect the cellular mechanism of rTMS. sAP is a transient depolarization of the membrane potential, which have the ability to convey primary rapidly signals and initiate cellular events, and the ability to show the small changes of input signals [28,29]. sAP is a complex process, generated by several special types of ion channels, including calcium channel, sodium channel, potassium channel, and so on [30]. Thus, the electrophysiology properties of sAP relate to the excitability of neurons and functions of the ion channels. In our study, to compare with the control group, the frequency of sAP from acute group was decreased significantly, the 12-h group was increased, however the 24-h group was kept unchanged (Figure 4). On the contrary, the resting potential of sAP from the acute group was increased, the 12-h group was decreased. In Figure 5, we observed that the calcium channel current has been influenced (Figure 5). Similar with the frequency of sAP, the current density and rise time of calcium channel from acute group have been increased, and the current density and rise time of calcium channel from 12-h group have been decreased. These changes in calcium channel were consistent with the present conclusion that rTMS regulated the pre-synapses of synapses by calcium channel as calcium channel is necessary for neurons to release neurotransmitters. Interestingly, our data presented the acute and chronic effects of rTMS on neural circuit, showing that rTMS generated acute inhibition effect on pre-synapses of synapses and excitation effect on pre-synapses 12 h after stimulation, however, these effects can disappear 24 h after rTMS, as rTMS regulated the neural circuit activity in a time-dependent manner. This rTMS-reduced short-term effect can be caused by the activity of calcium channel. Therefore, in order to release the long-term effects of rTMS, further studies associated with rTMS and calcium channel are needed. 

## 4. Material and Methods

### 4.1. Animals

Fly stock was raised on standard cornmeal/agar food, with 12:12-h light: dark cycles at 24 °C and 60% relative humidity, using standard *Drosophila* laboratory protocols [31]. Wild-type Canton-S fly pupae, 3 days before eclosion, were selected and placed in a new dish with standard food. The fly pupae were divided into control, 1 Hz-rTMS, 2 Hz-rTMS, 5 Hz-rTMS, 10 Hz-rTMS to detect the effects of rTMS at different frequency, with 100% intensity, the fly pupae were divided into control, 1%-rTMS, 10%-rTMS, 50%-rTMS, and 100%-rTMS groups for detecting the effects of rTMS at 1 Hz, with varying intensities, and the fly pupae were divided into control, acute, 12-h, and 24-h groups to detect the chronic effects of rTMS. For the chronic experiment, after rTMS, the 12-h and 24-h flies were raised in standard conditions before patch-clamp recordings. The flies were identified by red eyes and transparent wings in the puparium; *n* = 6 per group.

### 4.2. Application of rTMS

rTMS was performed with Magstim Rapid2 repetitive magnetic stimulator devices (Rapid2, Magstim, UK), with a maximum intensity of 2.3 T and stimulation frequencies from 1 Hz to 100 Hz. For rTMS, a 70-mm figure-8 Magstim coil was held 1 cm over the flies during the stimulation. The intensities, including 1%, 10%, 50%, and 100% of the maximum intensity of 2.3 T, were applied. Six pules of RTMS (pules train consisting of a pule with an inter-train interval of 30 s), lasting 5 s, was delivered. First, to estimate the effects of rTMS at different frequencies on synaptic plasticity, rTMS at varying frequencies (1 Hz, 2 Hz, 5 Hz, 10 Hz), with 100% intensity were delivered (showed as 1 Hz-rTMS, 2 Hz-rTMS, 5 Hz-rTMS, and 10 Hz-rTMS). Then, to estimate the effects of rTMS with different intensities at low-frequency (1 Hz), mEPSC were performed in the application of 4 intensities of rTMS, including 1%, 10%, 50% and 100% (showed as 1%-rTMS, 10%-rTMS, 50%-rTMS and 100%-rTMS) to estimate the acute effects of rTMS on synaptic plasticity. Finally, to detect the chronic impacts of rTMS, 1%-rTMS was applied to the flies. The electrophysiology properties, including mEPSC, sAP, and calcium channel currents of PNs were recorded immediately, 12 h after and 24 h after rTMS (showed as acute, 12-h and 24-h). 

### 4.3. Electrophysiology Experiments

The entire fly brain, including antennal lobes, was resected in standard external solution containing 20 units/mL papain with 1mM l-cysteine previously described [32]. Then the dissected brains were mounted in an RC-26 prefusion chamber (Warner Instruments, Hamden, CT, USA), containing a standard external solution. The standard external solution contained (in mM) 101 NaCl, 1 CaCl_2_, 4 MgCl_2_, 3 KCl, 5 glucose, 1.25 NaH_2_PO_4_, and 20.7 NaHCO_3_, pH 7.2, 250 Osm, and bubbled with 95% O_2_ and 5% CO_2_ (2 mL/min). The PNs were recorded with pipettes (10–15 MΩ) filled with an internal solution. The pipettes were pulled by a Flaming-Brown electrode puller (P-97; Sutter Instruments, Novato, CA, USA) using a 4-stage pull protocol.

Giga-ohm seals were achieved prior to recording in an on-cell configuration, followed by whole cell recordings in the standard external solution. For spontaneous action potential (sAP) and mini excitatory postsynaptic current (mEPSC) recordings, the pipette electrodes were filled with a standard internal solution containing (in mM): 140 potassium gluconate, 5 NaCl, 2 MgATP_2_, 1 CaCl_2_, 10 EGTA, and 10 HEPES (pH: 7.2–7.4). Under current-clamp configuration, the sAP was recorded, and only overshooting action potentials more positive than 0 mV were selected. The number of sAP, average resting potential (RP), frequency, amplitude, afterhyperpolarization, and time to maximum were analyzed. Under voltage-clamp configuration, the mEPSC were recorded with the membrane potential holding at −70 mV. A total of 1 μM tetrodotoxin (TTX) and 10 μM picrotoxin (PTX) were added into the external bath solution to block the voltage-gated sodium currents and γ-aminobutyric acid-ergic current respectively. mEPSC amplitudes < 20 pA and mEPSC frequency, calculated by counting the amplitude and frequency of sEPSCs occurrence within 2 min, were detected. For acute experiment, average frequency and amplitude of mEPSC were analyzed. For chronic experiment, average frequency, amplitude, rise time, and decay time of mEPSC were analyzed. For calcium channel recordings, 1 μM TTX, tetraethylammonium (TEA; 10 mM) and 4 aminopyridine (4-AP; 1 mM) were added to the external solution and cesium (Cs^+^; 102 mM) to the internal solution. A voltage pulse was applied, consisting of a 100 ms step to 40 mV from −70 mV (in 10 mV intervals) in voltage-clamp configuration. The current density was fit using an equation pA/pF = I_max_/Cs, where I_max_ is the peak current of calcium channel, and Cs is the series capacitance. Recordings were made at room temperature, and a single PN was examined in each brain (*n* = 6 per group). 

All electrophysiological recordings were performed using a BX51WI upright microscope (Olympus, Lehigh Valley, PA, USA). Signals were acquired by EPC10 amplifier (HEKA Elektronik, Lambrecht/Pfalz, Germany), and filtered at 5 kHz using a built-in filter and digitized at 5 kHz. Data analysis was performed by the pClamp10 Clampfit software (Molecular Devices, Sunnyvale, CA, USA).

### 4.4. Biocytin Staining and Confocal Image of Neuron

For biocytin staining, 0.4% biocytin was added into the internal solution. The soma and terminals were injected with the biocytin in the recording pipette in the whole cell configurations for at least 30 min. Then, the brain was fixed in phosphate buffered 4% formaldehyde at 4 °C for 3 h. Next, the brain was washed three times with 1% PBS, blocked and incubated in blocking buffer (0.1 MPBS, 0.1% Triton X-100, 1% BSA) containing streptavidin-CY3 (Molecular Devices) for 3 h at room temperature. After incubation, the brain was washed three times with PBS. ABX51WI microscope with a 40× objective and confocal camera was used to acquire photos of dendritic arborization of the visual projection neurons. Each representative image was randomly sampled ten times and the counter was blinded to sample identities (fly genotype, age, and other experimental conditions). 

### 4.5. Data Analysis

Values are presented as the mean ± SEM. Additionally, *p* < 0.05 was considered to be significant. Statistical analysis was conducted using SPSS 19.0 software (IBM SPSS, Armonk, NY, USA). All statistical analyses of the biological data were performed using t-tests, with the exception that the K-S test was used to perform the analysis of the cumulative probability data.

## 5. Conclusions

In summary, this study revealed that rTMS altered the electrophysiological properties of PNs from *Drosophila* brain. Moreover, rTMS affected the presynaptic properties of synapses in a frequency- and intensity-dependent manner and regulated the calcium channel activities in a time-dependent manner, revealing the neurological basis of the modulation mechanisms for rTMS. More interestingly, we found that the properties of mEPSC, sAP, and calcium channel currents from 24-h group have returned to average level, suggesting that the regulation of rTMS should be short-term effect. Thus, future studies should be performed, including the behavior test and genetic testing to deeply explore the effects of rTMS on structural neural plasticity and provide further insights into the cellular mechanisms involved in rTMS.
